# Distinguishing Between Biological and Technical Replicates in Hypertension Research on Isolated Arteries

**DOI:** 10.3389/fmed.2019.00126

**Published:** 2019-06-20

**Authors:** Dmitry Tsvetkov, Evgeniy Kolpakov, Mario Kassmann, Rudolf Schubert, Maik Gollasch

**Affiliations:** ^1^Experimental and Clinical Research Center, A Joint Cooperation Between the Charité Medical Faculty and the Max Delbrück Center for Molecular Medicine (MDC), Berlin, Germany; ^2^Department of Pharmacology and Experimental Therapy, Institute of Experimental and Clinical Pharmacology and Toxicology, Eberhard Karls University Hospitals and Clinics and Interfaculty Center of Pharmacogenomics and Drug Research, University of Tübingen, Tübingen, Germany; ^3^DZHK (German Centre for Cardiovascular Research), Partner Site Berlin, Berlin, Germany; ^4^Centre for Biomedicine and Medical Technology Mannheim and European Center of Angioscience, Research Division Cardiovascular Physiology, Medical Faculty Mannheim of the University Heidelberg, Mannheim, Germany; ^5^Department of Physiology, Medical Faculty, Augsburg University, Augsburg, Germany; ^6^Medical Clinic for Nephrology and Internal Intensive Care, Charité University Medicine, Berlin, Germany

**Keywords:** adipocyte-derived relaxing factor (ADRF), perivascular adipose tissue (PVAT), biological replicates, technical replicates, experimental study design, sample size, power of statistical analysis, hierarchical model

## Abstract

Perivascular adipose tissue (PVAT) is implicated in the pathophysiology of cardiovascular disease, especially in obese individuals in which the quantity of renal and visceral PVAT is markedly increased. The control of arterial tone by PVAT has emerged as a relatively new field of experimental hypertension research. The discovery of this prototype of vasoregulation has been mostly inferred from data obtained using wire myography. Currently, there is a major discussion on distinguishing between biological vs. technical replicates in biomedical studies, which resulted in numerous guidelines being published on planning studies and publishing data by societies, journals, and associations. Experimental study designs are determined depending on how the experimentator distinguishes between biological vs. technical replicates. These definitions determine the ultimate standards required for making submissions to certain journals. In this article, we examine possible outcomes of different experimental study designs on PVAT control of arterial tone using isolated arteries. Based on experimental data, we determine the sample size and power of statistical analyses for such experiments. We discuss whether *n*-values should correspond to the number of arterial rings and analyze the resulting effects if those numbers are averaged to provide a single *N*-value per animal, or whether the hierarchical statistical method represents an alternative for analyzing such kind of data. Our analyses show that that the data (logEC_50_) from (+) PVAT to (–) PVAT arteries are clustered. Intraclass correlation (ICC) was 31.4%. Moreover, it appeared that the hierarchical approach was better than regular statistical tests as the analyses revealed by a better goodness of fit (v^2^-2LL test). Based on our results, we propose to use at least three independent arterial rings from each from three animals or at least seven arterial rings from each from two animals for each group, i.e., (+) PVAT vs. (–) PVAT. Finally, we discuss a clinical situation where distinguishing between biological vs. technical replicates can lead to absurd situations in clinical decision makings. We conclude that discrimination between biological vs. technical replicates is helpful in experimental studies but is difficult to implement in everyday's clinical practice.

## Introduction

The control of arterial tone by perivascular adipose tissue (PVAT) has emerged as a relatively new field of vascular biology. After a pioneering report by Soltis and Cassis (1), several subsequent studies demonstrated a paracrine role for PVAT to produce relaxation of arterial smooth muscle cells (VSMC). This led to the discovery of a number of perivascular relaxation factors (PVRFs) and their downstream K^+^ channel targets (2, 3) to underlie arterial adipose-vascular coupling (4). The discovery of this prototype of vasoregulation has been mostly inferred from data obtained using wire myography on isolated arterial rings (5–10). Renal PVAT alters also renal vascular function, in particular because adipose tissue adjacent to renal blood vessels, i.e., renal perivascular adipose tissue (RPVAT), contains a pool of norepinephrine which can be released to alter renal vascular function and could contribute to renovascular hypertension (11). However, the further validation of novel concepts of preclinical research and their transfer into clinical practice can face a crucial obstacle observed in many other areas of scientific practice: irreproducibility.

As such, reproducibility in biomedical research has become an important topic of debate in the scientific community (12, 13). Some data have indicated that only 20 % of studies published even in prestige journals can be replicated (14). To address this problem, the scientific community undertakes a lot of efforts to improve the quality of animal studies, which resulted in numerous guidelines on study planning, statistical evaluation, and data presentation published by societies, journals, and associations (15, 16). Some of these recommendations include but are not limited to: blinding of experiments, randomized allocation of animals to control and treatment groups, training in and more appropriate use of statistics, use of appropriate positive, and negative controls; determination of dose-response relationships, and replication in different models, as reviewed in (17–19).

Although recommendations toward the design of biomedical studies are widespread and supported by pharmacological societies (16), they still need consideration. One particularly troubling aspect is distinguishing between biological vs. technical replicates (20). Experimental study designs are determined depending on how the experimentator distinguishes between biological (considered “independent”) vs. technical replicates. These definitions govern the ultimate standards required for making submissions to certain journals. In addition, they are expected to have an impact on decision-making by scientific journals, funding agencies, authorities, and offices of animal welfare. Generally, biological replicates are defined as measurements of biologically distinct samples that show biological variation (21). In contrast, technical replicates are repeated measurements of the same sample that show independent measures of the noise associated with the equipment and the protocols. According to these definitions, it is not entirely clear to which group isolated arteries belong to? This issue is not as simple as it seems. For example, if we consider well-known regional differences of vessel function and the recently emphasized fact that even cells in a small segment of an artery show large differences in behavior (22, 23). Is this a result of independent samples? Moreover, calculations of sample size, power and effect size are often missing (or at least not reported) for measurements obtained using wire myography (one of the “gold standard” methods in vascular research on arterial tone), including studies with a focus on detecting anti-contractile effects of PVAT.

In the past, studies in the field of PVAT research used standard statistical tests to detect differences between data sets [e.g., (+) PVAT vs. (–) PVAT]. Those tests (e.g., Mann-Whitney U or *t*-test) are valid for independent samples. As a sample, one can chose one piece of artery (*n* = number of arteries), independent on whether they have been obtained from the same or different animals. Alternatively, one can take *n* arteries from *N* animals, calculate a mean for the *n* arteries obtained from one and the same animal and treat the *N* animals as independent samples. The last approach is less common in practice, because it decreases the (apparent) ability to detect a difference, takes more efforts and finally could be considered as breaking one of the 3R principles in animal research—Reduction (24).

In this article, we examine possible outcomes of different experimental designs addressing as an example PVAT control of arterial tone. Based on previously collected data, we also determined the required sample size for appropriate statistical analysis for such experiments. We discuss whether *n*-values should correspond to the number of vessels and analyze the resulting effects if those numbers are averaged to provide a single value per animal, or whether the hierarchical statistical method represents an alternative for analyzing such kind of data.

## Methods

**Figure 1:**
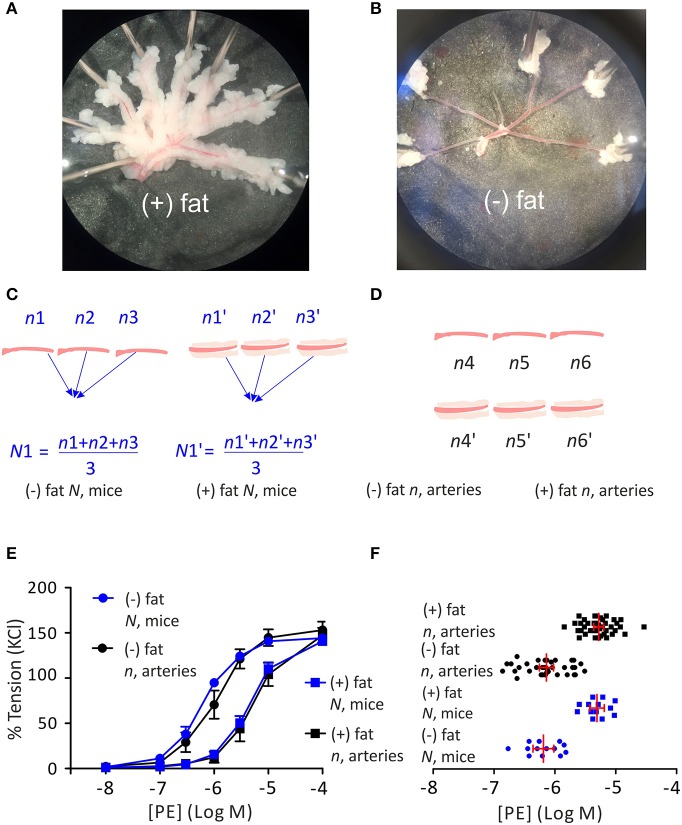
Regulation of arterial tone by perivascular adipose tissue (PVAT). First order mesenteric arteries, perivascular fat and connective tissue were either intact [(+) PVAT **(A)** or removed (–) PVAT **(B)**]. Schematic representation of using the average per animal (*N*, mice) approach **(C)** or single artery (*n*, arteries) approach **(D)** for PVAT studies. Cumulative concentration-response relationships to phenylephrine (PE) with *n* representing the number of arteries or *N* representing the number of mice **(E)** expressed as a percentage of KCl-induced contraction. Data are mean ± SE. (–) PVAT, *n*, arteries: *n* = 10; (+) PVAT, *n*, arteries: *n* = 10. (–) PVAT, *N*, mice: *N* = 10; (+) PVAT, *N*, mice: *N* = 10. LogEC_50_ for PE between (–) PVAT and (+) PVAT groups **(F)** analyzed either as *n*, arteries (–) PVAT, *n* = 38; (+) PVAT, *n* = 39 or *N*, mice (–) PVAT, *N* = 13; (+) PVAT, *N* = 13. Data are mean ±95% Cl. *P* < 0.05, ANOVA with *post-hoc* Tukey test *N*, mice (–) PVAT vs. *N*, mice (+) PVAT and *n*, arteries (–) PVAT vs. *n*, arteries (+) PVAT.

All experimental procedures were performed in accordance with the German legislation on protection of animals. Animal care followed American Physiological Society guidelines and local authorities (Landesamt für Gesundheit und Soziales Berlin, LAGeSo) approved all protocols. Mice (C57BL/6) were housed in individually ventilated cages under standardized conditions with an artificial 12-h dark–light cycle with free access to water and food. Wire Myography on first order mesenteric arteries was performed as previously described (25, 26). First order mesenteric arteries were dissected into 2 mm rings whereby perivascular fat and connective tissue were either intact [(+) PVAT (Figure 1A) or removed (–) PVAT (Figure 1B)] without damaging the adventitia. The symbol *n* represents the number of independent arteries tested (e.g., *n* = 10: artery 1, 2, 3, 4, 5, 6, 7, 9, 10). Data from multiple rings from the same animal were averaged and treated as a single *N* [e.g., *N* = 3: (artery 1, 2, 3), (artery 4, 5, 6), (artery 7, 8, 9)]. Quantile-quantile (q-q) plot and a Shapiro-Wilk test were used to test the normality of the data distribution. For deeper analysis, anaconda 4.4.0 was used, including the following software: python v3.6.1, matplotlib v2.0.2, numpy v1.13.1, pandas v0.20.3, and statsmodels v0.8.0. Concentration-response fitting and EC_50_ calculation were performed using scipy.optimization.curve_fit routine assuming Log-logistic 4-parameter function curve, independently for each artery/animal. Effect size and sample size calculations on EC_50_ values for a two-sided *t*-test were performed using G^*^ power software v3.1.9.3 (27). Statistical significance was determined by two-tailed *t*-test or ANOVA with the *post-hoc* Tukey test. For the calculations based on hierarchical analysis we used RStudio v1.1.383 and the R-script as previously described (28). v^2^ test of change in −2 Log Likelihood (v^2^-2LL) was used for the comparison of the hierarchical model vs. regularly used statistical tests (29, 30). Effective sample size was calculated as follows: for *N* animals, each with *n* arteries, and ICC representing intraclass correlation.

$$\begin{array}{l} Effective\;Sample\;Size\;=\frac{N\;\times \;n}{1+\left(n-1\right)\times ICC} \end{array}$$

## Results

### Replicate Number *n* (Single Artery) vs. *N* (Averaged per Animal)

First, we determined whether using *N* (averaged per animal) as shown in Figure 1C vs. *n* (single artery) as shown in Figure 1D provides advantages for detecting the anti-contractile effect of PVAT using wire myography. For that reason, from a total data set of 13 mice we randomly assigned similar numbers of arteries (*n* = 10) and animals (*N* = 10). There were totally 10 arteries (isolated from 3 mice), that were treated as independent samples; or totally 38 arteries isolated from 10 mice, whereas the data were averaged per animal and treated as independent sample *N*. We found that both methods gave similar results, as can be seen in the graphical analysis (Figure 1E).

In a detailed analysis, we evaluated the LogEC5_50_ values calculated either based on the *n* (single arteries) or the *N* (averaged per animal) approach (Figure 1F). The number of arteries collected from one animal ranged from two to five with a mean value of 3 in both the (+) PVAT and (–) PVAT groups. The LogEC_50_ 95% CI for PE were −6.02 to −6.26 and −6.02 to −6.36 for the (–) PVAT group treated as single arteries or averaged per animal, respectively; and −5.20 to −5.36 and −5.19 to −5.43 for the (+) PVAT group treated as single arteries or averaged per animal, respectively.

### Two-Level Hierarchical Model for Detecting the Anti-contractile Effect of PVAT

To evaluate clustering of our data, we estimated the intraclass correlation (ICC) also known as the degree of clustering. ICC quantifies the magnitude of clustering. The ICC was 31.4 %. The hierarchical model resulted in a better fit (v^2^ test of change in −2 Log Likelihood; see also methods) than the regularly used method [single artery (*n*) or average per animal (*N*) approach] for data analysis, although both methods resulted in successfully detecting the difference between the (+) PVAT and (–) PVAT groups (Table 1).

**Table 1:** Analysis of the α1-receptor mediated contraction of (+) PVAT and (–) PVAT arterial rings using regular (*N* or *n* approach) and hierarchical statistical approaches.

**Parameter**	**Clustering of data (ICC), %**	**Regular method**		**Two-level hierarchy**		**Comparison of goodness of fit (regular vs. hierarchical)**
		**Standard error**	***P*-value**	**Standard error**	***P*-value**	
PE LogEC_50_	31.4	0.068	<0.05	0.087	<0.05	0.0094

*The independent t-test represents the regular approach to compare contraction of (+) PVAT and (–) PVAT arterial rings. The clustering of the data was quantified by the intraclass correlation coefficient (ICC). Comparison of goodness of fit was performed by the v^2^-2LL test*.

### Required Sample Size in PVAT Studies

Next, we calculated the effect size (Cohen's d) of the difference between the PE concentration response relationships of vessels with and without PVAT (based on EC_50_), i.e., the anti-contractile effect of PVAT. Using the obtained effect size, we also estimated the minimal sample size required for detecting the PVAT effect with a power of 0.9 (1—type II error or beta) at different levels of type I error or alpha (*p* = 0.05, *p* = 0.01, *p* = 0.001). Using the hierarchical approach as previously described (28), we also calculated the effective sample size for this experimental design. The ICC can be used to estimate the effective sample size that is neither *n* nor *N*. If the ICC is low, meaning each artery's behavior is independent of the animal from which the artery was isolated, effective sample size approximates:

$$\begin{array}{l} Effective\;Sample\;Size\;=\frac{N\;\times \;n}{1+\left(n-1\right)\times ICC} \end{array}$$

**Figure 2:**
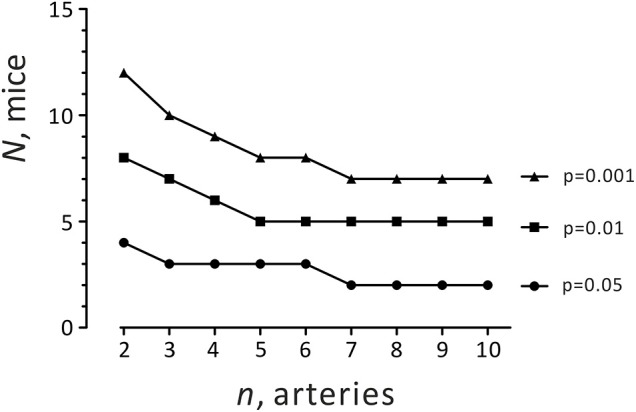
Required sample size (different combinations of *n* and *N*) for detecting the anti-contractile effect of PVAT using the hierarchical approach.

If the ICC is high, meaning behavior of arteries from a single animal is identical, effective sample size approaches *N*. The results show that for detecting PVAT-induced relaxation at *p* = 0.05 using the hierarchical approach, the effective sample size is equal to 6. The data are summarized in Figure 2 and Table 2.

**Table 2:** Required sample size for detecting the anti-contractile effect of PVAT.

	***n*****, arteries (single artery approach)**				***N*****, mice (average per animal approach)**				**Hierarchical approach**
	**(–) PVAT**	**(+) PVAT**	**Effect size**	**Sample size (*n*)**	**(–) PVAT**	**(+) PVAT**	**Effect size**	**Sample size (*N*)**	**Effective sample size**
PE LogEC_50_	−6.14 ±0.35	−5.28 ±0.26	2.81	4^*^ 6^**^ 9^***^	−6.19 ±0.28	−5.31 ±0.20	3.62	3^*^ 5^**^ 7^***^	6^*^ 12^**^ 18^***^

The effect size of the difference in the means (logEC_50_ for PE) between (–) PVAT and (+) PVAT arterial rings is expressed as Cohen's d. Sample size was calculated based on the use of the t-test. There were (–) PVAT, n = 38; (+) PVAT, n = 39 arteries; N = 13 mice in (–) PVAT and (+) PVAT groups. Sample size is given for

* *p = 0.05*,

** *p = 0.01*,

*** *p = 0.001. p—Type I error or alpha. Power (1—Type II error or beta) is 0.9*.

## Discussion

Whilst in the past many important discoveries in medicine and biology have been made without profound statistical analysis (31), it is remarkable that nowadays the effect size seems to be in focus of interest to contribute to major a key role in discoveries. In contrast to basic research, size effect in clinical trials is typically small implicating that thousands of participants are often needed to be included in order to make reliable conclusions (32). Of note, the effect size for PVAT-induced relaxation can be classified as relatively large (33). Based on this effect size, relatively small numbers of experiments are expected to be needed to be performed in order to detect the anti-contractile effect of PVAT (Table 2). However, there remains a fundamental question, which needs to be considered: What is the most appropriate strategy to design and analyze studies focused on PVAT function in myography experiments on isolated arteries? In other words, what is the independent sample criterion? According to Blainey et al., one strategy is that organs from sacrificed animals can be viewed as biological replicates (21). However, it is not possible to view all arteries in an organism as a single organ. Large caliber vessels differ from small caliber vessels. Cerebral arteries differ from skeletal muscle or renal arteries in the organism. In particular, cerebral arteries cannot exhibit an anti-contractile effect of PVAT because PVAT does not surround brain vessels, and thus, they have different mechanisms of arterial tone regulation.

Biological variation, however, is also defined as variation between organisms. In the past, researches used two different approaches for analyzing data in PVAT studies. First, a more conservative approach also known as the average per animal approach was employed. According to this strategy, all measurements—data points—obtained from a certain intervention from several similar objects (e.g., several pieces of a mesenteric artery) of one animal are averaged per animal and treated as a single (*N*) data point, as shown on Figure 1C. It is obvious that this approach requires relatively large numbers of animals to test biological hypotheses. Treating samples as *N* (per animal) reduces the standard deviation (SD) and thus standard error of the mean (SEM) in wire myography experiments compared to the single artery approach (Figures 1E,F). As a result, the effect size (Cohen's d) of the difference of EC_50_ between (–) PVAT and (+) PVAT using the average per animal approach is larger than with single artery approach (details see below). Although the average per animal approach results in relatively small sample sizes required to detect the anti-contractile effect of PVAT (Table 2), the number of experiments to be performed (number of vessels studied) is relatively high. In fact, instead of using nine arteries from one or two mice we would need to use seven mice to detect the same difference (Table 2). Therefore, the average per animal approach might come into conflict with one of the most important foundations of the 3Rs principle of animal research—Reduction (24) without revealing a different conclusion (Figures 1E,F). On the other hand, this strategy may decrease the probability of false positive or negative findings due to a “poor preparation day.”

For a clinician, implementing the average per subject approach to a patient with peripheral artery disease might also be problematic. For example, in peripheral artery disease (also called peripheral arterial disease) there is circulatory problem in which narrowed arteries reduce critical blood flow, e.g., to fingers or legs. If the clinical diagnoses shows critical digital ischemia in one of the patient's fingers what surgical treatment (amputation) is appropriate? One might argue that data from multiple arteries/cells from the same organism should be averaged and treated as a single N, which would be the inappropriate approach. The situation might be even more complicated if two fingers show 100% stenosis (occlusion) with gangrene and amputations are not considered as *n* = 2 independent surgeries, i.e., disaggregation of *n* = 2 arteries but rather aggregation of *N* = 1 human. These examples demonstrate how essential it is to distinguish between biological (sometimes called “independent”) vs. technical replicates in daily clinical decision-making.

**Figure 3:**
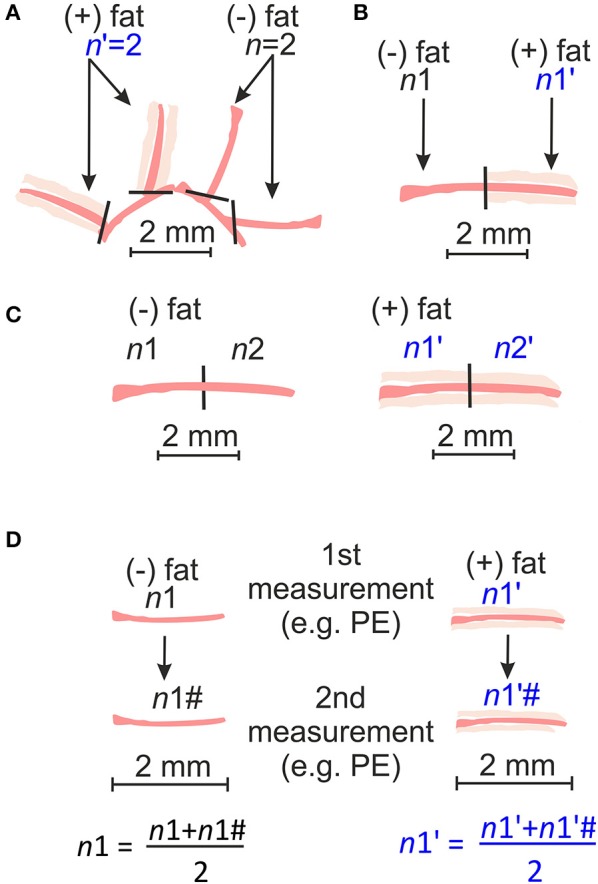
Schematic representation of arterial rings obtained from independent arteries dissected into 2 mm long rings **(A)** or obtained from the same arteries but prepared either with (+) PVAT or without (–) PVAT **(B)**; or obtained from the same artery and prepared in the same way [(–)PVAT, (+) PVAT] **(C)**; or technical replicate whereas the contractility of the same arterial ring is measured twice and then averaged and treated as single *n* **(D)**.

The second strategy is most commonly used in experimental research on isolated arteries and is also called the single artery approach. This method assumes the independency of each data point *n* (artery) and views an artery as source of the biological variation (Figure 1D). In this view, isolated mesenteric arteries are considered as objects of the study. A current scheme for conducting such experiments is shown in Figure 3A. Independently, 1-st order branches of mesenteric arteries should be mounted on the wire myograph either with (+) PVAT or without (–) PVAT. The scheme shown in Figures 3B,C also could be used, although there is no data available neither confirming nor disproving any meaningful physiological or molecular-biological differences between these closely related segments. In the scenario shown in Figure 3D, it is obvious that the same segments are measured twice in the same chamber and therefore should be viewed as a technical replicate. In this case, data obtained from two measurements should be averaged and treated as single *n* as it is recommended in Curtis et al. (16).

It is also reasonable to argue that arteries isolated from the same location in the circulatory system and from the same animal will tend to behave similarly. For this situation, a special statistical method has been developed, namely the hierarchical analysis (29, 30). According to the two-level hierarchical model, data points in a cluster (e.g., arteries from the same animal) tend to be more similar to each other compared to data points in other clusters (arteries from different animals). Recently, Sikkel et al. successfully implemented this method for analyzing local Ca^2+^ image data (Ca^2+^ sparks) in rats and concluded that Ca^2+^ sparks in rat cardiomyocytes are clustered (28). Therefore, the assumption of the independency of each data point can lead to the false conclusion about a difference (28). We tested this approach and found that the hierarchical method is superior to previously described average per animal or single artery approaches for wire myography experiments in the absence or presence of PVAT (Table 1). Indeed, we found that our data (logEC_50_) are clustered (ICC 31.4%). Moreover, it appeared that the hierarchical approach was better than regular statistical tests as the goodness of fit (v^2^-2LL test) for the hierarchical approach was better. In addition, we calculated the effective sample size for our PVAT studies (Table 2). For detecting a PVAT-induced relaxation at *p* = 0.05 using the hierarchical approach, the effective sample size was equal to 6. The results suggest to use at least two arterial rings from each from four animals for each group, i.e., (+) PVAT vs. (–) PVAT. To minimize the number of animals used, three arterial rings from each from three animals or at least seven arterial rings from each from two animals. This conclusion is based on an effective sample size calculation for hierarchical analysis (Figure 2 and Table 2).

In summary, the experimental design of biomedical studies is still a matter of intense ongoing discussions in renovascular hypertension research. We examined three approaches to be used in statistical analyses of data on PVAT control of arterial tone. We conclude that neither the *N* (averaged per animal) nor the *n* (single artery) approach provides an optimal statistical tool for detecting the anti-contractile effect of PVAT using wire myography. Both approaches have advantages and limitations as discussed above. We conclude that hierarchical analysis of data points seems to represent a reasonable approach to draw reliable conclusions. The strength of our study lies within the statistical treatment of the data and the way that we have considered the variation in the biological assays. However, whether data obtained in other vascular studies are also clustered remains to be evaluated. For example, it needs to be studied whether our approach can be implemented in video microscopic studies using pressurized arteries or patch clamp studies on isolated cells. However, due to technical reasons, it would be very difficult to investigate PVAT effects in smaller pressurized vessels. In this regard, the fat tissue surrounding vessels is expected to perturb measurements of the inner and outer diameters of the vessels. Nevertheless, our approach could be used for the design of future studies using isolated arteries in wire-myography experiments. In addition, we estimated the minimal sample size required for detecting PVAT-induced relaxations using the two-level hierarchical method, which can be used for studies in the research on arterial adipose-vascular coupling and in experimental hypertension research. Based on our results, we propose that usage of at least three arterial rings each from three mice is a reliable approach for wire myography to study PVAT control of arterial tone. Furthermore, hierarchical statistical analysis represents a better alternative statistical tool for PVAT studies. In comparison to other research areas (e.g., clinical trials), this sample size seems to be relatively small. The explanation for this state of affairs is the relatively large size effect of PVAT on arterial tone, which underscores and highlights the pivotal role of PVAT in this relatively novel prototype of vasoregulation.

## Author Contributions

DT and MK performed the wire myography experiments. DT and EK performed statistical analysis. DT drafted the article. DT, EK, MK, RS, and MG planned and designed the experimental studies and contributed to its completion.

### Conflict of Interest Statement

The authors declare that the research was conducted in the absence of any commercial or financial relationships that could be construed as a potential conflict of interest.
